# Unhealthy weight control behaviors and health risk behaviors in American youth: a repeated cross-sectional study

**DOI:** 10.1186/s40337-024-01081-1

**Published:** 2024-08-19

**Authors:** Yunan Zhao, Alvin Tran, Heather Mattie

**Affiliations:** 1 Department of Population Health, NYU Grossman School of Medicine, New York, NY USA; 2 Department of Population Health and Leadership, University of New Haven, West Haven, CT USA; 3 Department of Biostatistics, Harvard T.H. Chan School of Public Health, Boston, MA USA

**Keywords:** Unhealthy weight control behaviors (UWCBs), Body mass index (BMI), Youth risk behavior surveillance system (YRBSS), Self-perceived weight, Self-perceived weight bias

## Abstract

**Background:**

Unhealthy weight control behaviors (UWCBs) involve weight control strategies to reduce or maintain weight, such as fasting, taking diet pills, and vomiting or taking laxatives. UWCBs in teenagers can escalate into severe health issues such as eating disorders. Understanding the trends of UWCBs and their association with risk behaviors in teenagers is crucial, as early intervention and prevention strategies are pivotal.

**Methods:**

This study utilized eight waves of the youth risk behavior surveillance system (YRBSS) data from 1999 to 2013. Our primary outcome was UWCBs engagement. We used multinomial logistic models to analyze the association between UWCBs and risk behaviors among adolescents including driving after alcohol consumption, suicide attempts, smoking, alcohol use, and sexual intercourse.

**Results:**

Among 109,023 participants, UWCBs prevalence was 16.64%. Body Mass Index (BMI) was significantly associated with UWCBs risk. In addition, we found the intention of weight management confounded the relationship between BMI and UWCBs. The unadjusted logistic regression indicated a monotone-increasing association between BMI and the risk of UWCBs. In contrast, the adjusted logistic regression indicated a U-shaped curve with the lowest (BMI < 17 kg/m^2^) and highest (BMI > 30 kg/m^2^) BMI groups having significantly higher odds of engaging in UWCBs compared to the reference BMI group (18.5 ≤ BMI ≤ 24.9 kg/m^2^).

**Conclusions:**

The intention of weight management confounded the relationship between Body Mass Index (BMI) and the risk of UWCBs. These findings suggest that healthcare interventions for weight management behaviors should be tailored to adolescents with BMI ≥ 25 and BMI < 18.5.

**Supplementary Information:**

The online version contains supplementary material available at 10.1186/s40337-024-01081-1.

## Abstract (plain language summary)

This study looked at unhealthy weight control behaviors (UWCBs) in American teenagers, such as fasting, taking diet pills, or vomiting to control weight. These behaviors can lead to serious health problems, including eating disorders. The research analyzed data from over 100,000 teenagers between 1999 and 2013 to understand the connection between UWCBs and other risky behaviors like drinking alcohol, smoking, and attempting suicide. While previous research suggested that teens with higher body weight were more likely to engage in UWCBs, our findings showed a shift in this relationship after considering the teens’ intentions to manage weight. Specifically, teens with very low body weight (BMI < 17) and a strong desire to lose weight had an increased risk of UWCBs. This highlights the need for healthcare providers to focus on the mental health and weight management goals of these teens to prevent harmful behaviors. Additionally, reconsidering the removal of UWCB-related questions from national surveys like YRBSS is important to continue monitoring these behaviors.

**Supplementary Information:**

The online version contains supplementary material available at 10.1186/s40337-024-01081-1.

## Background

Western society exhibits a preference for a thin body shape [6]. The current standard of “the ideal body” portrayed in the media is slimmer than it has been in the past, reinforcing the internalization of the societal thin beauty ideal [22]. Simultaneously, comprehensive prejudices against individuals living in larger bodies persist, fuelled by weight-based stereotypes that label such individuals as lazy, greedy, and lacking self-discipline [14]. Sociocultural pressures arouse concerns over body image, particularly among adolescents who strive to meet the social beauty ideal [10]. Consequently, weight control has become an indispensable aspect of their daily life.

Previous research suggests weight loss holds benefits for overweight and obese individuals, especially for insulin-resistant and hyperinsulinemic patients [16]. However, the approach to weight control is important as adolescents often adopt a spectrum of approaches. Unhealthy Weight Control Behaviors (UWCBs), such as fasting (not eating for 24 or more hours), taking diet pills, and vomiting or taking laxatives, have significant negative effects on adolescents from both physical and psychological perspectives [21]. Persistent engagement in UWCBs may lead to a number of adverse outcomes, such as tooth enamel erosion, gastroesophageal reflux [17], 23], and potential effects of melanosis coli resulting from irritant purgatives [18]. Furthermore, UWCBs are frequently deemed as proxy risk factors for eating disorders, signifying a greater probability of psychological impairment, suicide attempts, and mortality from psychiatric syndromes without appropriate intervention [20]. Understanding the trends of UWCBs in young people and identifying their risk factors are crucial, as they allow experts to both evaluate existing efforts and propose new interventions to address this public health issue.

One potential influencing factor of UWCBs is body mass index (BMI). The prevailing view proposes that adolescents with higher BMIs have a higher probability of engaging in UWCBs. For example, one study investigated risk factors associated with UWCBs using data from the Youth Risk Behavior Surveillance System (YRBSS) in 2013 [5, 25]. This study explored associations with various factors, including suicidal behaviors, driving after alcohol consumption, and physical education class attendance. This study also examined the relationship between weight status and UWCBs. The study’s findings suggest that adolescents with BMIs greater than 25 demonstrated a 3.6-fold higher likelihood of engaging in UWCBs compared to those with lower BMIs. Similarly, another study on body change strategies among Spanish adolescents indicated that elevated weight status increases the risk of UWCBs [1]. Both studies categorized BMI into three categories: BMI < 18.5, BMI within 18.5–24.9, and BMI ≥ 25. While these cutoff points assumed a constant BMI effect within each weight status, they raised questions about the variability of UWCB risk within the same weight status level. For instance, the likelihood of UWCB engagement for BMI < 17 may differ from that of those with BMI between 17 and 18.5. Although previous studies have shown which risk factors should be included in statistical models, the broad classification of weight states should be reconsidered. Apart from focusing on the weight management behaviors of people with BMI ≥ 25, future studies should also examine the weight management strategies of people with BMI < 18.5.

Previous studies have also explored the associations between UWCBs and other health risk behaviors in adolescents. Research analyzing data from the National Longitudinal Study of Adolescent Health (Add Health) identified gender differences in the likelihood of UWCBs, such as purging and diet pill use [19]. Add Health followed more than 20,000 adolescents over 14 years with four waves of data collection and collected information on UWCBs from 1994 to 2002. Research findings suggested that UWCBs were significantly associated with higher body mass index, self-perception of being overweight, low self-esteem, depression, and delinquency. Another study also investigated differing risk factors for UWCBs by sex and weight status [11]. Despite relying on nationally representative longitudinal cohort data, these studies faced limitations in measuring UWCBs. The Add Health questionnaire only asked participants to report the UWCBs within a seven-day timeframe, which may not fully capture the prevalence of these behaviors. As UWCBs occurring beyond the one-week snapshot would not be accounted for, this could underestimate their overall prevalence.

In addition to comparing the results of participants with different weight statuses and risk behaviors, calls for more comprehensive research that examines changes of UWCBs over time stem from the recognition that cross-sectional studies often dominate the current landscape [2]. As responses from the questionnaire in the Add study only provide information at the individual level, comparing the results across time could be informative for identifying trends, patterns, and evolving dynamics at the societal level. Dianne et al. investigated how the trends in disordered eating developed from 1999 to 2010 [12]. Using the pooled data from YRBSS, the article examined the prevalence of obesity and the UWCBs among different sex groups. The study used inverse probability weighting to account for socio-demographic changes over time, indicating a decreasing trend in UWCBs among adolescent girls. Using data from 1999 to 2013 Massachusetts YRBSS, Watson et al. [24] corroborated the finding that the prevalence of disordered eating behaviors decreased. Both studies shed light on encouraging results regarding the decreasing trend of UWCBs.

This study aims to extend the research in this area by examining the UWCBs-BMI link across different groups of U.S. adolescents from 1999 to 2013. It acknowledges previous studies analyzing the association between UWCBs and BMI, such that the probability of engaging in UWCBs increased for adolescents with BMI ≥ 25. However, the weight management strategies of people with BMI < 18.5 are virtually unexplored, and thus, the assumption of an association that is monotonically increasing with BMI should be questioned. Additionally, our study investigates whether UWCBs connected to self-perceived weight status and self-perceived weight bias. We hypothesize that the odds of engaging in UWCBs do not monotonically increase with the BMI of U.S. adolescents. We also hypothesize that self-perceived weight status could contribute to the risk of UWCBs for U.S. adolescents.

## Methods

The data for this study were sourced from the YRBSS, accessible via the Centers for Disease Control and Prevention (CDC). This study examined YRBSS data from 1999 to 2013, as records of weight management were not assessed after 2013 [5]. Over this period, the number of states conducting YRBSS varied from 37 to 47, and the sample size across states differed based on weighted population estimates. Participants completed a structured survey consisting of demographic inquiries, followed by sections addressing risk behaviors associated with UWCBs and then behaviors and thoughts related to weight management. The current study excluded participants under 14 years old due to the small sample size and a large percentage of missingness for BMI and attempted UWCBs.

We examined associations between unhealthy weight control behaviors and selected risk behaviors categorized into four domains: (1) unintentional injuries and violence (driving after alcohol consumption, suicide attempts); (2) substance use (smoking status, current alcohol use, marijuana use); (3) sexual behavior (sexual intercourse, condom usage); and (4) physical inactivity (physical education class attendance) [11, 12, 19, 25]. We utilized the World Health Organization (WHO) BMI cut-off points, classifying participants into five levels: level 1 (BMI < 17 kg/m^2^), level 2 (17.0 ≤ BMI ≤ 18.49 kg/m^2^), level 3 (18.5 ≤ BMI ≤ 24.9 kg/m^2^), level 4 (25 ≤ BMI ≤ 29.9 kg/m^2^), and level 5 (BMI ≥ 30 kg/m^2^) [26]. We selected BMI 18.5–24.9 kg/m^2^ (level 3) as the reference group and created indicator variables for the other categories. We utilized numeric BMI categories considering concerns around obesity stigma [15].

The YRBSS questionnaire asked participants to self-report their current engagement in fasting, taking diet pills, and vomiting within the past 30 days, with the response options for all three questions being binary, indicating “yes” or “no” for current engagement in each behavior. The dependent variable “UWCBs” was generated using the results of these three variables to indicate whether the participant engaged in any of the three types of UWCBs within the past 30 days. To determine whether the missing data of UWCBs was related to variables of interest, we fit a logistic regression model regressing the missing indicators of UWCBs on BMI group, self-perceived weight, and intention of weight management, respectively.

In addition, the study included “self-perceived weight bias” based on BMI and “self-perceived weight” to study whether a participant’s self-perceived weight category aligned with their calculated BMI category. Self-perceived weight was assessed in the YRBSS questionnaire using the prompt, “How do you describe your weight?” with five response choices including: (1) very underweight; (2) slightly underweight; (3) about the right weight; (4) slightly overweight; and (5) very overweight. The variable “self-perceived weight bias” was derived as a binary variable, integrating both “self-perceived weight” and BMI categories. Specifically, if individuals’ perceived weight did not align with their observed weight, “self-perceived weight bias” was coded as nonzero.

To evaluate the association between UWCBs and the BMI group, we fit multiple logistic regression models. We first developed Model 1, adjusting only for the survey year and BMI group. Due to the non-linear relationship between years and UWCBs, we employed spline models to capture the flexibility in the relationship between years and UWCBs. Model 2 included the variables in Model 1 and adjusted for additional variables, including age, sex, race/ethnicity, driving after alcohol consumption, suicide attempts, smoking status, current alcohol use, and sexual intercourse. Model 3 included all variables in Model 2 and an extra variable related to how individuals manage their weight, categorized into four levels: (1) lose weight; (2) gain weight; (3) stay the same weight; and (4) not trying to do anything. To avoid collinearity in our models, we constructed a correlation matrix for both risk behavior variables and potential confounding variables associated with BMI and UWCBs. We also conducted sensitivity analyses to test the robustness of our findings. We fit models using data from each year individually in addition to an overall model that pooled data from all years and was adjusted for each year.

The data cleaning process included using the SPSS syntax and the ASCII data files provided on the CDC website, editing the SPSS files according to the instructions, and converting the ASCII data files into a permanent SPSS data files that include labels and formats. The R programming language version 4.2.2 was used to import the SAV file and create the subset of the original data. *P* values < 0.05 were considered statistically significant.

## Results

The final sample included 109,023 participants (Table 1). Of the participants, 50.3% were female (*n* = 54,789), 46.2% were White (*n* = 50,341), and 22.2% were Black or African American (*n* = 24,218). For BMI categories, 9.8% had a BMI > 30 (*n* = 10,687), and 2.1% had a BMI < 17 (*n* = 2267). In the study, 16.6% (*n* = 18,097) of participants reported engaging in UWCBs, 82.3% (*n* = 89,715) reported not engaging in UWCBs, and 1.1% (*n* = 1,211) did not specify. Based on the analysis for missing data, the UWCB’s missingness did not have a significant relationship between BMI group (*p* = 0.819), self-perceived weight (p = 0.485), and intention of weight management (*p* = 0.414). Since the missingness was independent of variables of interest, we concluded that the missing values in UWCBs were missing at random.

**Table 1:** Descriptive statistics of participants by BMI level

	Level 1 (N = 2267)	Level 2 (N = 7081)	Level 3 (N = 68,993)	Level **4** (N = 19,995)	Level 5 (N = 10,687)	Overall (N = 109,023)
*Sex*						
Female	1241 (54.7%)	4197 (59.3%)	35,542 (51.5%)	9095 (45.5%)	4714 (44.1%)	54,789 (50.3%)
Male	1026 (45.3%)	2884 (40.7%)	33,451 (48.5%)	10,900 (54.5%)	5973 (55.9%)	54,234 (49.7%)
*Age (years)*						
14	402 (17.7%)	1087 (15.4%)	6397 (9.3%)	1468 (7.3%)	675 (6.3%)	10,029 (9.2%)
15	706 (31.1%)	2058 (29.1%)	15,827 (22.9%)	3960 (19.8%)	1989 (18.6%)	24,540 (22.5%)
16	570 (25.1%)	1777 (25.1%)	17,964 (26.0%)	4986 (24.9%)	2634 (24.6%)	27,931 (25.6%)
17	404 (17.8%)	1435 (20.3%)	18,130 (26.3%)	5696 (28.5%)	3082 (28.8%)	28,747 (26.4%)
18	185 (8.2%)	724 (10.2%)	10,675 (15.5%)	3885 (19.4%)	2307 (21.6%)	17,776 (16.3%)
*Race*						
White	1186 (52.3%)	3759 (53.1%)	33,169 (48.1%)	8187 (40.9%)	4040 (37.8%)	50,341 (46.2%)
Black or African American	353 (15.6%)	1151 (16.3%)	14,577 (21.1%)	5027 (25.1%)	3110 (29.1%)	24,218 (22.2%)
Hispanic or Latino	320 (14.1%)	1040 (14.7%)	11,746 (17.0%)	4165 (20.8%)	2208 (20.7%)	19,479 (17.9%)
Asian	170 (7.5%)	385 (5.4%)	2558 (3.7%)	479 (2.4%)	171 (1.6%)	3763 (3.5%)
Multiple—Non-hispanic	89 (3.9%)	268 (3.8%)	2352 (3.4%)	630 (3.2%)	347 (3.2%)	3686 (3.4%)
Multiple—Hispanic	50 (2.2%)	182 (2.6%)	1822 (2.6%)	536 (2.7%)	306 (2.9%)	2896 (2.7%)
Am Indian/Alaska Native	56 (2.5%)	138 (1.9%)	1310 (1.9%)	514 (2.6%)	272 (2.5%)	2290 (2.1%)
Native Hawaiian/other PI	23 (1.0%)	98 (1.4%)	943 (1.4%)	303 (1.5%)	156 (1.5%)	1523 (1.4%)
Missing	20 (0.9%)	60 (0.8%)	516 (0.7%)	154 (0.8%)	77 (0.7%)	827 (0.8%)
*Driving after alcohol* consumption						
Yes	277 (12.2%)	925 (13.1%)	11,337 (16.4%)	3512 (17.6%)	1857 (17.4%)	17,908 (16.4%)
No	1920 (84.7%)	5998 (84.7%)	56,013 (81.2%)	15,910 (79.6%)	8505 (79.6%)	88,346 (81.0%)
Missing	70 (3.1%)	158 (2.2%)	1643 (2.4%)	573 (2.9%)	325 (3.0%)	2769 (2.5%)
*Suicide attempt*						
Yes	402 (17.7%)	1168 (16.5%)	10,565 (15.3%)	3317 (16.6%)	2052 (19.2%)	17,504 (16.1%)
No	1853 (81.7%)	5879 (83.0%)	57,967 (84.0%)	16,559 (82.8%)	8555 (80.1%)	90,813 (83.3%)
Missing	12 (0.5%)	34 (0.5%)	461 (0.7%)	119 (0.6%)	80 (0.7%)	706 (0.6%)
*Smoke (past 30 days)*						
Yes	911 (40.2%)	3254 (46.0%)	35,854 (52.0%)	11,185 (55.9%)	6228 (58.3%)	57,432 (52.7%)
No	1232 (54.3%)	3519 (49.7%)	30,135 (43.7%)	7998 (40.0%)	3960 (37.1%)	46,844 (43.0%)
Missing	124 (5.5%)	308 (4.3%)	3004 (4.4%)	812 (4.1%)	499 (4.7%)	4747 (4.4%)
*Alcohol (past 30 days)*						
Yes	664 (29.3%)	2574 (36.4%)	28,485 (41.3%)	8213 (41.1%)	4227 (39.6%)	44,163 (40.5%)
No	1444 (63.7%)	4065 (57.4%)	35,870 (52.0%)	10,208 (51.1%)	5507 (51.5%)	57,094 (52.4%)
Missing	159 (7.0%)	442 (6.2%)	4638 (6.7%)	1574 (7.9%)	953 (8.9%)	7766 (7.1%)
*Sexual behavior*						
Yes	723 (31.9%)	2712 (38.3%)	33,349 (48.3%)	10,340 (51.7%)	5179 (48.5%)	52,303 (48.0%)
No	1379 (60.8%)	3884 (54.9%)	30,708 (44.5%)	8200 (41.0%)	4652 (43.5%)	48,823 (44.8%)
Missing	165 (7.3%)	485 (6.8%)	4936 (7.2%)	1455 (7.3%)	856 (8.0%)	7897 (7.2%)
*Weight management*						
Lose weight	280 (12.4%)	1033 (14.6%)	25,659 (37.2%)	13,643 (68.2%)	8389 (78.5%)	49,004 (44.9%)
Gain weight	872 (38.5%)	2396 (33.8%)	14,286 (20.7%)	1392 (7.0%)	336 (3.1%)	19,282 (17.7%)
Stay the same weight	433 (19.1%)	1659 (23.4%)	14,967 (21.7%)	2534 (12.7%)	797 (7.5%)	20,390 (18.7%)
Not trying to do anything	658 (29.0%)	1922 (27.1%)	13,246 (19.2%)	2166 (10.8%)	1022 (9.6%)	19,014 (17.4%)
Missing	24 (1.1%)	71 (1.0%)	835 (1.2%)	260 (1.3%)	143 (1.3%)	1333 (1.2%)
Attempting *UWCBs*						
Yes	243 (10.7%)	702 (9.9%)	10,133 (14.7%)	4280 (21.4%)	2739 (25.6%)	18,097 (16.6%)
No	2006 (88.5%)	6322 (89.3%)	58,082 (84.2%)	15,472 (77.4%)	7833 (73.3%)	89,715 (82.3%)
Missing	18 (0.8%)	57 (0.8%)	778 (1.1%)	243 (1.2%)	115 (1.1%)	1211 (1.1%)

BMI Level: Level 1 (BMI < 17 kg/m^2^), Level 2 (BMI: 17.0–18.49 kg/m^2^), Level 3 (BMI: 18.5–24.9 kg/m^2^), Level 4 (BMI: 25–29.9 kg/m^2^), Level 5 (BMI ≥  30 kg/m^2^)

**Fig. 1:**
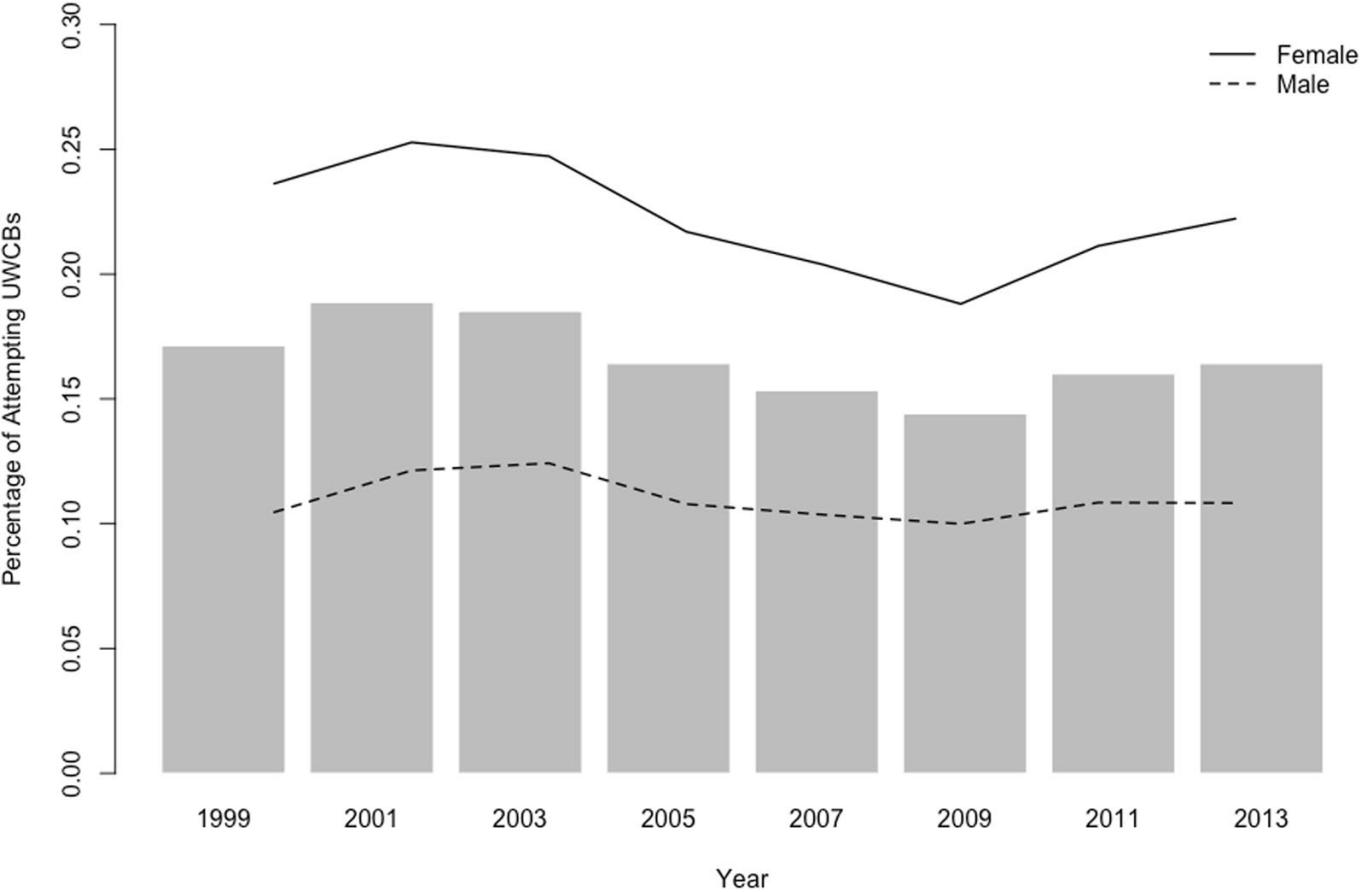
Prevalence of Unhealthy Weight Control Behaviors (UWCBs) by sex over time

Prevalence of engaging in UWCBs across survey years is displayed in Fig. 1. Female participants exhibited a higher rate of engaging in UWCBs (*Mean* = 22.23%, *SD* = 2.21%) than male participants (*Mean* = 10.98%, *SD* = 0.85%). The estimated risk of UWCBs associated with age increased by 0.61% per year (*p* < 0.001), with females having a 10.96% greater risk than males (*p* < 0.001).

**Fig. 2:**
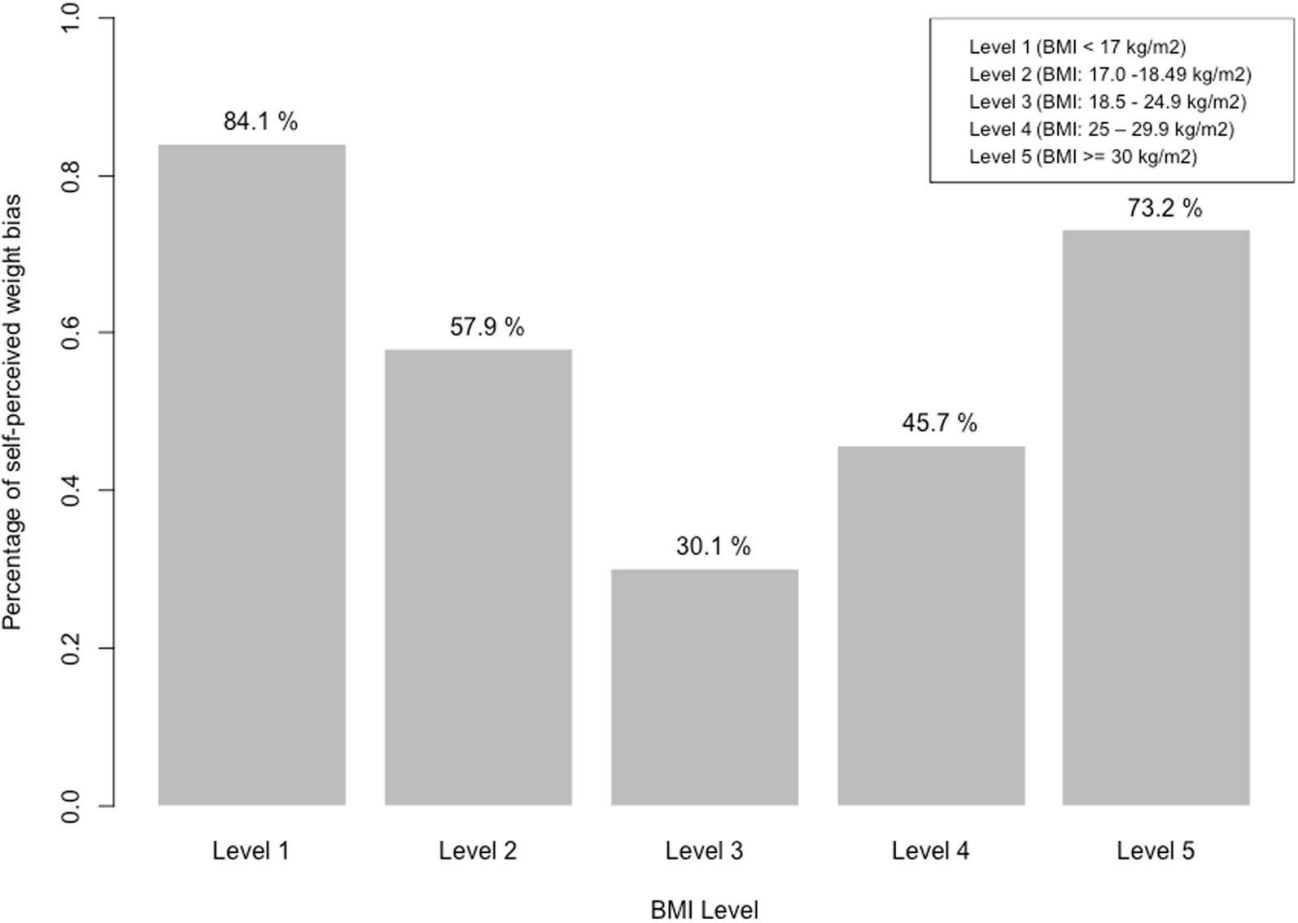
Self-perceived weight bias by BMI level

The proportion of self-perceived weight bias was 40.1%. Of participants, the proportion of mis-perceivers was 73.2% for BMI > 30 kg/m^2^ and 84.1% for participants with BMI < 17 kg/m^2^. The trend revealed a higher proportion of participants with self-perceived weight bias existed in BMI levels 1 and 5 (Fig. 2). When investigating the self-perceived weight bias between different BMI groups, this particular trend remained from 1999 to 2013 as we stratified the data by year.

**Fig. 3:**
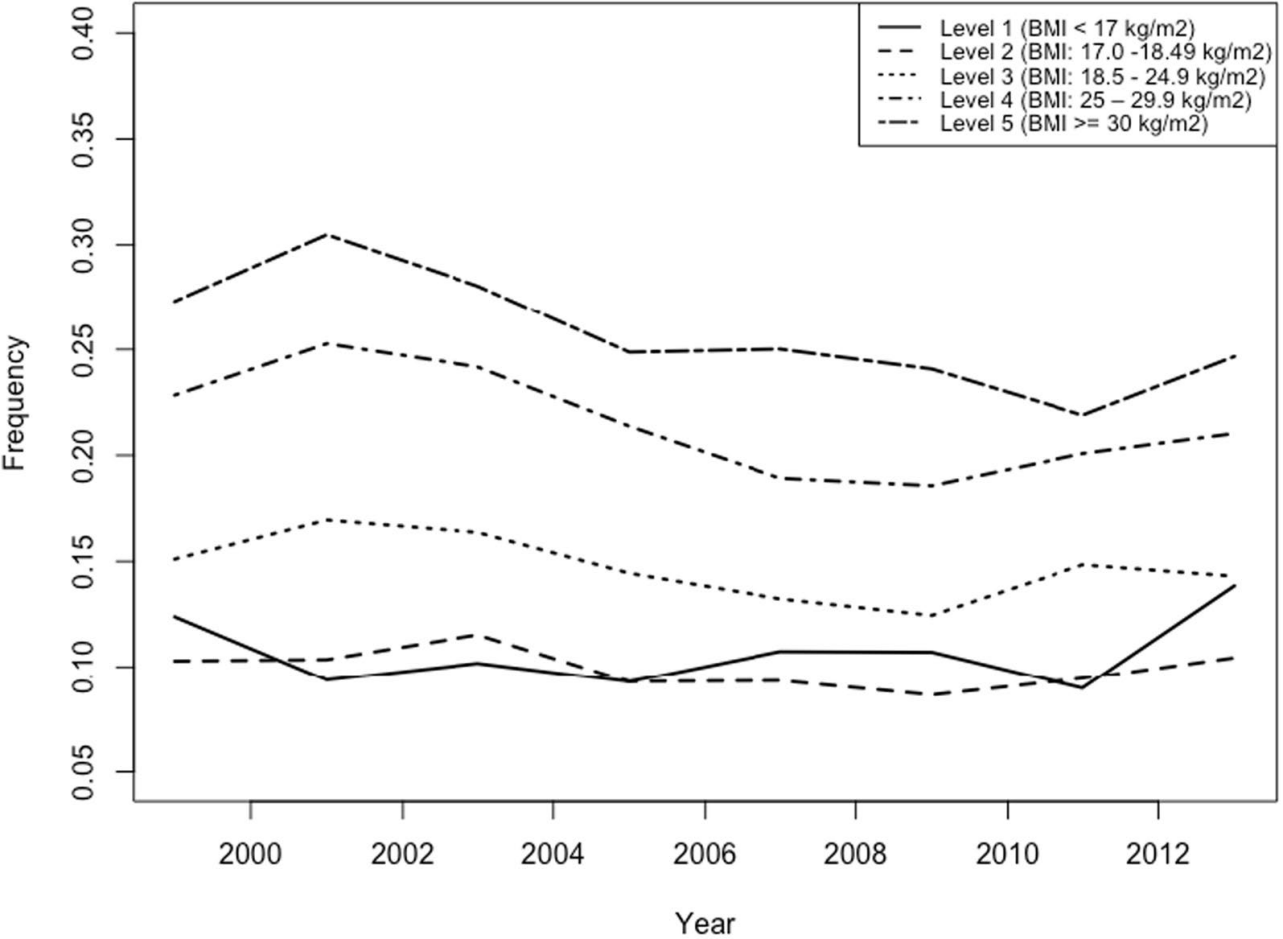
Prevalence of UWCBs

The prevalence of engaging in UWCBs was lower for participants with BMI level 1 and BMI level 2 compared to the baseline group, and the prevalence of UWCBs was greater for those with BMI level 4 and BMI level 5 (Fig. 3). In Model 1, adjusted only for survey year and BMI, the odds of UWCBs was smaller for participants with BMI level 1 compared to the reference group, BMI level 3 (Odds Ratio [95% CI]: 0.69 [0.60, 0.79], *p* < 0.001; see Table 2). Conversely, the estimated odds ratios were elevated for groups with a BMI greater than the reference group (level 3), particularly BMI level 5 (Odds Ratio [95% CI]: 2.02 [1.92, 2.12], *p* < 0.001). In Model 2, adjusted for survey year, BMI, demographics, and selected risk behaviors, the odds of engaging in UWCBs were lower for groups with a BMI lower than the reference group (see Table 2).

**Table 2:** The association between UWCBs for participants with different BMI groups

	Model 1		Model 2		Model 3	
	Odds ratio [95% CI]	*p* value	Odds ratio [95% CI]	*p* value	Odds ratio [95% CI]	*p* value
BMI Level 1	0.69 [0.60, 0.79]	< 0.001	0.69 [0.59, 0.79]	< 0.001	1.16 [0.99, 1.34]	0.056
BMI Level 2	0.63 [0.58, 0.69]	< 0.001	0.58 [0.54, 0.64]	< 0.001	0.93 [0.85, 1.02]	0.107
BMI Level 4	1.59 [1.53, 1.66]	< 0.001	1.72 [1.65, 1.79]	< 0.001	1.12 [1.07, 1.18]	< 0.001
BMI Level 5	2.02 [1.92, 2.12]	< 0.001	2.20 [2.09, 2.32]	< 0.001	1.29 [1.22, 1.36]	< 0.001

Model 1, year adjusted; Model 2, adjusting year, demographics and selected risk behaviors; Model 3, adjusting year, demographics, selected risk behaviors and weight management

Reference group for each model: BMI Level 3 (18.5–24.9 kg/m^2^); Demographics covariates include age, sex, and race/ethnicity; selected risk behaviors included driving after alcohol consumption, suicide attempts, smoking status, current alcohol use, and sexual intercourse

**Fig. 4:**
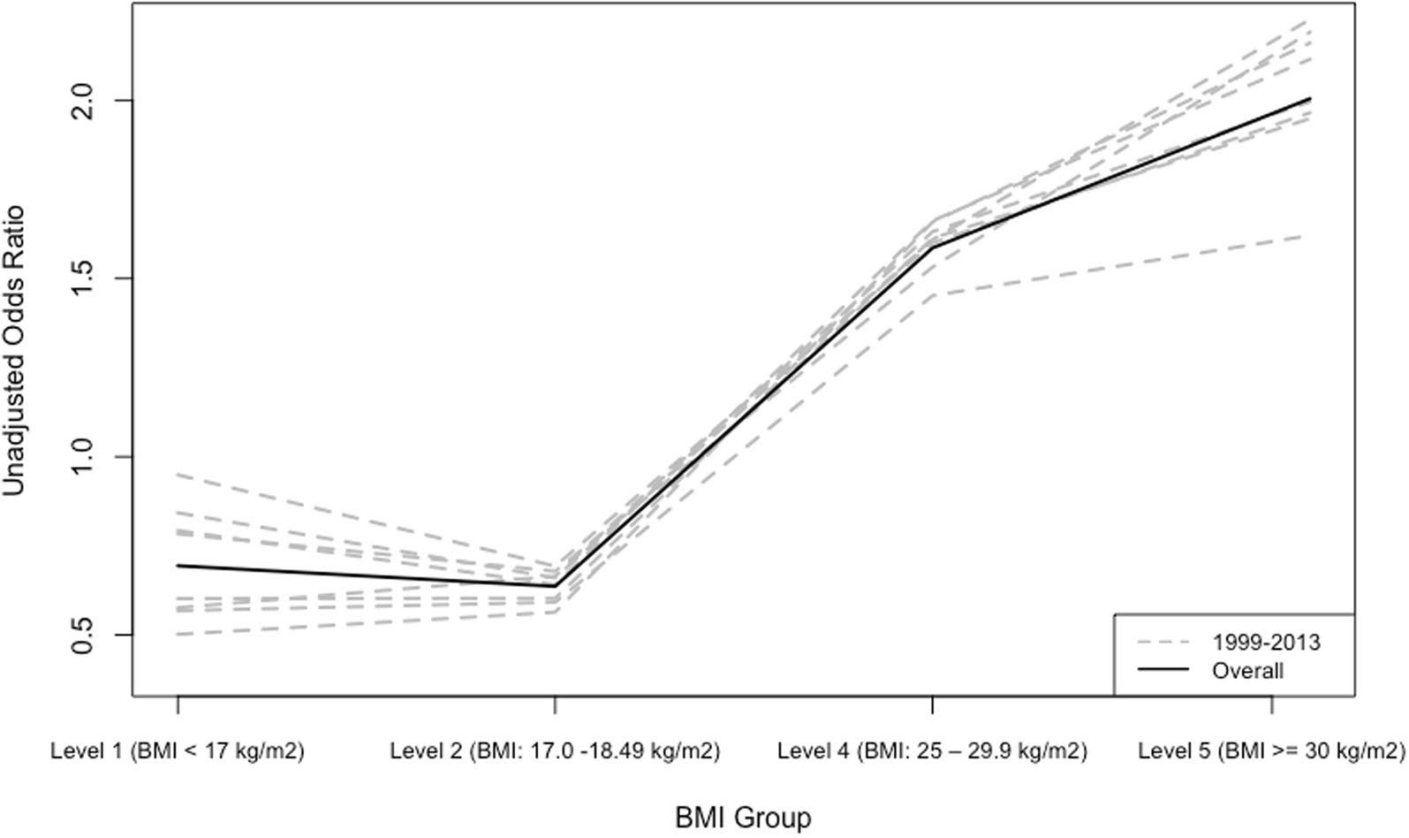
Unadjusted ORs of UWCBs

**Fig. 5:**
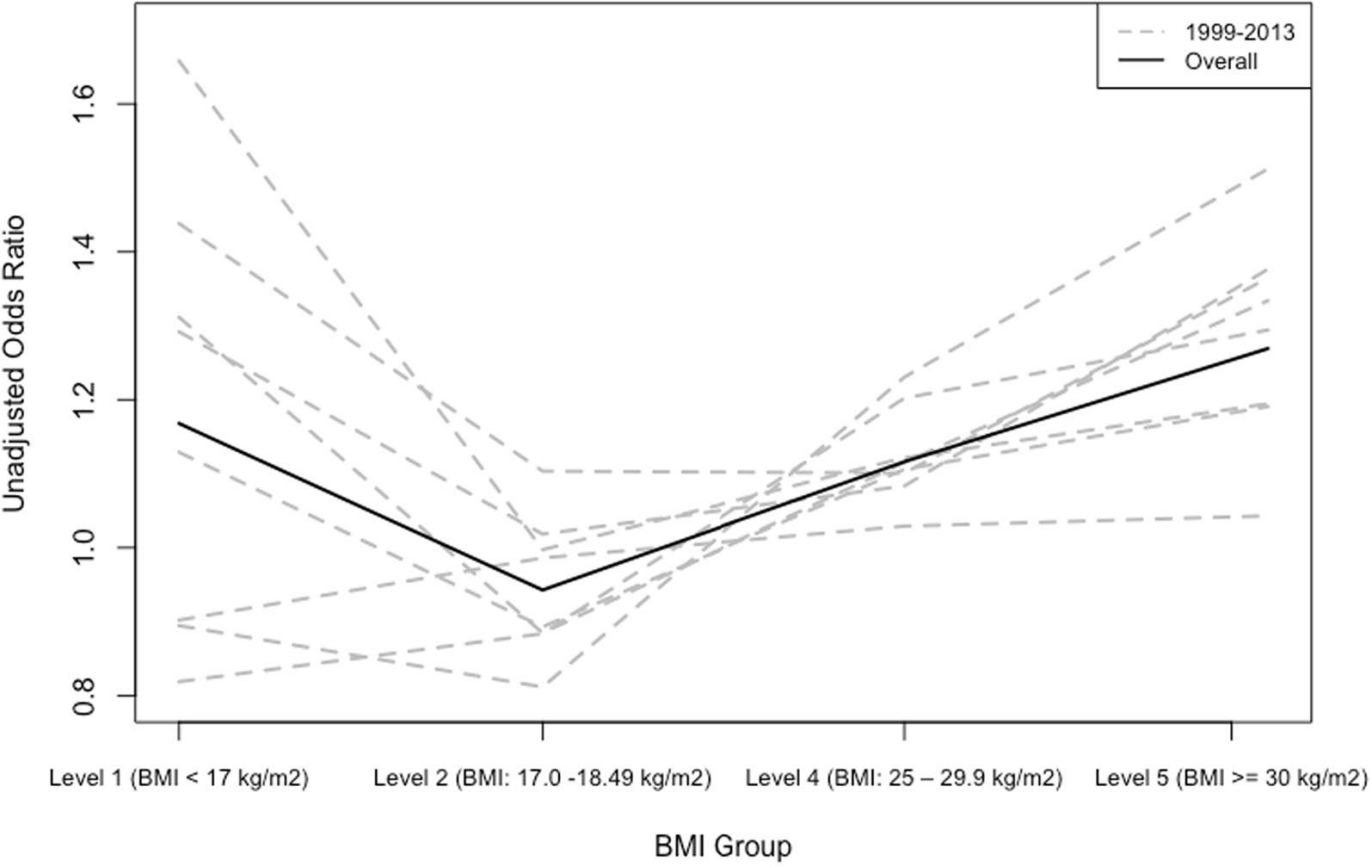
Adjusted ORs of UWCBs

The inference from Models 1 and 2 is not valid if known confounding factors are not controlled for. According to the correlation matrix, presented in Supplementary Table 1, “weight management” is negatively correlated with both BMI (r = 0.30) and UWCBs (r = − 0.27), and the correlations were much stronger than other variables used in the models. Considering the causal directed acyclic graph (DAG) association between BMI and UWCBs, the intention of weight management is a common cause of both BMI group and UWCBs status. Thus, we examined the relationship between BMI and the likelihood of UWCBs, controlling for weight management in Model 3. The relationship between BMI and the risk of UWCBs shifted from a monotonically increasing curve to a U-shaped curve (see Fig. 4 and Fig. 5). Estimated odds ratios for participants trying to lose weight, gain weight, and stay the same weight for all BMI groups were greater than the reference group who were not trying to do anything about their weight. The overall estimated OR of the UWCBs for group BMI level 1 is 1.16 (95% CI: [0.99, 1.34], *p* = 0.056), indicating a 15.5% higher likelihood than the reference group (see Table 2). Although the p-value slightly exceeded the 0.05 threshold, our analysis revealed that group BMI level 1 had higher odds of engaging in UWCBs than the reference group after adjusting for weight management based on the 95% confidence intervals, contradicting results from Model 1 and Model 2. In the sensitivity analysis to assess differences in adjusted odds ratio across different survey years, we found a similar pattern of changes in the relationship between BMI and the risk of UWCBs (Supplementary Table 2). The effect of weight management was strongest in the year 2013.

## Discussion

Our results suggest a significant association between sex and the overall differences in unhealthy weight control behaviors, with a notable observation of higher prevalence among females; the likelihood of performing UWCBs doubled as we stratified by male and female participants. Additionally, our analyses suggest that UWCBs were driven by BMI group. The association between BMI group and the likelihood of performing UWCBs changed significantly from Model 1 and Model 2 to Model 3, particularly after adjusting for weight management. In Model 1 and Model 2, participants in the BMI level 1 group were less likely to engage in UWCBs, and participants in the BMI level 5 group were more likely to perform UWCBs. However, the monotone linear increasing trend changed into a U-shaped trend after adjusting for participants’ intention of weight management in Model 3. Additionally, participants’ self-perceived weight bias had a high level of variation across different BMI groups.

Firstly, the current study explored Weng’s (2020) and Almenara’s [1] findings that body mass index predicts UWCBs. Previous studies suggested a positive correlation between higher BMI and increased likelihood of UWCBs among adolescents; Model 1 and Model 2 of the current study supported this relationship among high school students in the U.S. However, this finding did not persist in the confounder-adjusted Model 3. Notably, the study by Weng et al. (2020) considered weight loss intention and actual weight status in their multiple logistic regression model [25]. The weight status variable was recorded in three categories based on body mass index percentile for children and teens: underweight (< 5%), normal weight (5–84.99%), and overweight (≥ 85%).

Our findings replicated the findings of Stephen et al. [19] and Nagata et al. [11], revealing a greater prevalence of UWCBs among female participants compared to males. While the previous studies examined UWCBs in a seven-day timeframe, the questionnaire used for the current study asked participants to recall UWCBs from the past 30 days, which presented broader implications due to the extended timeframe. Despite the timeframe variation, both studies identified sex as a potential risk factor for UWCBs. In addition, the missingness of USCBs was not significantly correlated with BMI, which made the previous studies more conclusive [12, 24].

Together, our research mainly suggests that the intention of weight management might change the likelihood of high school students in the U.S. performing UWCBs stratified by BMI groups. Though concern for groups with BMI ≥ 25 was on the rise, people should offer similar concerns to students with BMI < 18.5 who intend to lose weight. There exists a more common eating disorder pathology in female athletes, especially in sports emphasizing leanness [8]. With lower BMIs on average, these young people are under extra pressure from their sports environment to maintain or lose weight [3]. Since body image anxiety brought up a prevalent concern, helping students raise their self-confidence about their body image may improve the lives of young people across the population. In addition to the regular educational forums on race discrimination and sex discrimination, schools should offer special information sessions for adolescents to emphasize not judging or bullying other people based on body image and body shape.

Additionally, the current study illustrated the importance of considering confounding bias in associational studies. The true association from the adjusted logistic model may be the opposite to the estimated association from the unadjusted model. Future studies could investigate BMI groups, weight management intention, and UWCBs more thoroughly among adolescents in different contexts, such as high schools in Asian countries.

The current study had several limitations. While the collected sample size of the questionnaire was satisfactory, the participants only reflected only unhealthy weight control behaviors in the United States. Further information is required for these results to be generalizable to other populations and countries. Additionally, since the YRBSS data collected after 2013 excluded the UWCBs section, the study only included data from 1999–2013, which did not represent the current UWCBs situation [5]. The findings should be contextualized within this timeframe. Since 2013, there have been notable shifts in societal attitudes towards weight and increased influence of digital media, which may impact the applicability of our results to today’s youth [9].

Also, the study relied on self-report questionnaires, introducing the challenge of socially desirable responses. Participants might tend to respond in a socially desirable manner, potentially leading to an underestimation of UWCBs. This study examined whether the missing responses in UWCBs were associated with participants’ intentions concerning social desirability response to address the issue of self-reported data. Among the 2000 participants who responded to part of the three UWCBs, 82% answered “No” to the question(s) regarding current UWCBs. Possibly, some participants were reluctant to admit to UWCBs. More information is required to understand the impact of socially desirable responses on the attitude of participants toward UWCBs. In this study, we assumed that the likelihood of performing UWCBs among non-responding participants was the same among respondents.

In addition, we conceptualized “weight status” with five categories based on the current WHO BMI cut-off for adults. While BMI serves as a reasonable indicator of body fat in both adults and children, general debates exist about its indirect measurement of body fat and its appropriateness as a diagnostic tool. Several studies propose using BMI percentiles to categorize different BMI groups, suggesting that this approach helped interpret a participant’s BMI relative to other teenagers of the same sex and age [7]. However, the available clinical growth charts from the CDC website, last reviewed on August 23, 2001, did not align with the BMI distribution in the current dataset [4]. Consequently, we utilized the adult BMI cut-off to establish a standardized framework for interpreting weight status.

In modern society, there is no shortage of information regarding healthy weight management. Yet, the bulk of research has shown that young people consistently engage in unhealthy weight control behaviors. Given the implications for physical and mental health, researchers need to understand the links between UWCBs and other risk behaviors. Notably, the removal of UWCB-related questions from the YRBSS questionnaire by the CDC in 2015 needs further discussion, considering the ongoing relevance of UWCBs in adolescent health research [5]. Additionally, researchers should pay attention to the mental health status of teenagers with lower BMIs who express the intention of weight loss. With the significant relationship between social media usage and body image issues, particularly among adolescents, social media platforms may contribute to heightened body dissatisfaction and disordered eating [13]. This growing concern highlights the urgency for more comprehensive studies and targeted interventions to evaluate and mitigate the negative impact of social media on young individuals’ self-image and mental health. Thus, we advocate for a careful assessment of adolescents with BMI < 18.5 who exhibit a preoccupation with weight management during clinical visits at healthcare institutions. This approach aims to prevent the risk of exacerbating unhealthy weight control behaviors and promote healthier outcomes for individuals who are overlooked.

### Supplementary Information

Additional file1

## Data Availability

The data analysed in the current study are available from the CDC YRBS website [www.cdc.gov/yrbs].
