# Supplementary material for: Unhealthy weight control behaviors and health risk behaviors in American youth: a repeated cross-sectional study
Source: J Eat Disord. 2024 Aug 19;12:118. doi: 10.1186/s40337-024-01081-1 (PMC11331811; doi:10.1186/s40337-024-01081-1)
Supplement: Supplementary file 1 — Additional file1 [file 40337_2024_1081_MOESM1_ESM.docx]

**ADDITIONAL FILE**

**SUPPLEMENTARY TABLE 1** Correlation Matrix for UWCBs Data

**SUPPLEMENTARY TABLE 2** The Association between UWCBs for Participants with Different BMI Groups for each Survey Year

**Supplemental Table 1** Correlation matrix for UWCBs data

|  | Age | Sex | Drive Drink | Suicide | Smoke | Alcohol | Marijuana | | Sexual | | Condom | PE | BMI | Weight Management | UWCBs |
| --- | --- | --- | --- | --- | --- | --- | --- | --- | --- | --- | --- | --- | --- | --- | --- |
| Age | 1.00 |  |  |  |  |  |  |  | |  | |  |  |  |  |
| Sex | 0.04 | 1.00 |  |  |  |  |  |  | |  | |  |  |  |  |
| Drive Drink | 0.16 | 0.11 | 1.00 |  |  |  |  |  | |  | |  |  |  |  |
| Suicide | 0.02 | 0.13 | -0.06 | 1.00 |  |  |  |  | |  | |  |  |  |  |
| Smoke | -0.14 | -0.03 | -0.18 | 0.13 | 1.00 |  |  |  | |  | |  |  |  |  |
| Alcohol | 0.13 | 0.01 | 0.31 | -0.13 | -0.40 | 1.00 |  |  | |  | |  |  |  |  |
| Marijuana | 0.17 | 0.07 | 0.25 | -0.13 | -0.53 | 0.45 | 1.00 |  | |  | |  |  |  |  |
| Sexual | -0.27 | -0.06 | -0.21 | 0.10 | 0.37 | -0.33 | -0.44 | 1.00 | |  | |  |  |  |  |
| Condom | 0.18 | -0.06 | 0.14 | -0.11 | -0.22 | 0.17 | 0.25 | -0.48 | | 1.00 | |  |  |  |  |
| PE | -0.23 | 0.10 | -0.03 | 0.03 | 0.03 | -0.02 | -0.03 | 0.05 | | -0.06 | | 1.00 |  |  |  |
| BMI | 0.12 | 0.07 | 0.03 | -0.03 | -0.07 | 0.02 | 0.04 | -0.05 | | 0.04 | | -0.03 | 1.00 |  |  |
| Weight Management | 0.00 | 0.19 | 0.00 | 0.10 | 0.06 | -0.06 | -0.03 | 0.02 | | -0.03 | | 0.01 | -0.03 | 1.00 |  |
| UWCBs | 0.01 | -0.17 | 0.08 | -0.23 | -0.14 | 0.15 | 0.12 | -0.09 | | 0.08 | | -0.03 | 0.12 | -0.27 | 1.00 |

**Supplemental Table 2** The association between UWCBs for participants with different BMI groups for each survey year

*Note:*

*Model 1, year adjusted;*

*Model 2, adjusting year, demographics and selected risk behaviors;*

| Survey Year: 1999 | | | | | | | |
| --- | --- | --- | --- | --- | --- | --- | --- |
|  | Model 1 | | Model 2 | | | Model 3 | |
|  | Odds Ratio  [95% CI] | p value | | Odds Ratio  [95% CI] | p value | Odds Ratio  [95% CI] | p value |
| BMI Level 1: | 0.79 [0.56, 1.10] | 0.18 | | 0.72 [0.49, 1.01] | 0.067 | 1.31 [0.89, 1.89] | 0.156 |
| BMI Level 2: | 0.64 [0.52, 0.79] | <0.001 | | 0.55 [0.44, 0.68] | <0.001 | 0.88 [0.70, 1.11] | 0.297 |
| BMI Level 4: | 1.66 [1.49, 1.85] | <0.001 | | 1.82 [1.62, 2.05] | <0.001 | 1.12 [0.98, 1.26] | 0.085 |
| BMI Level 5: | 2.12 [1.85, 2.42] | <0.001 | | 2.45 [2.12, 2.83] | <0.001 | 1.33 [1.14, 1.56] | <0.001 |

| Survey Year: 2001 | | | | | | | |
| --- | --- | --- | --- | --- | --- | --- | --- |
|  | Model 1 | | Model 2 | | | Model 3 | |
|  | Odds Ratio  [95% CI] | p value | | Odds Ratio  [95% CI] | p value | Odds Ratio  [95% CI] | p value |
| BMI Level 1: | 0.50 [0.33, 0.73] | <0.001 | | 0.47 [0.31, 0.70] | <0.001 | 0.89 [0.57, 1.35] | 0.612 |
| BMI Level 2: | 0.56 [0.45, 0.70] | <0.001 | | 0.48 [0.38, 0.60] | <0.001 | 0.81 [0.64, 1.03] | 0.088 |
| BMI Level 4: | 1.66 [1.48, 1.86] | <0.001 | | 1.90 [1.67, 2.14] | <0.001 | 1.23 [1.08, 1.40] | 0.002 |
| BMI Level 5: | 2.16 [1.88, 2.48] | <0.001 | | 2.54 [2.17, 2.96] | <0.001 | 1.51 [1.28, 1.78] | <0.001 |
| \| Survey Year: 2003 \| \| \| \| \| \| \| \| \| --- \| --- \| --- \| --- \| --- \| --- \| --- \| --- \| \|  \| Model 1 \| \| Model 2 \| \| \| Model 3 \| \| \| Odds Ratio  [95% CI] \| p value \| \| Odds Ratio  [95% CI] \| p value \| Odds Ratio  [95% CI] \| p value \| \| BMI Level 1: \| 0.58 [0.37, 0.86] \| 0.011 \| \| 0.53 [0.33, 0.81] \| 0.005 \| 0.82 [0.50, 1.29] \| 0.406 \| \| BMI Level 2: \| 0.66 [0.53, 0.82] \| <0.001 \| \| 0.59 [0.47, 0.74] \| <0.001 \| 0.88 [0.69, 1.12] \| 0.318 \| \| BMI Level 4: \| 1.63 [1.47, 1.81] \| <0.001 \| \| 1.83 [1.63, 2.06] \| <0.001 \| 1.20 [1.06, 1.36] \| 0.003 \| \| BMI Level 5: \| 2.00 [1.75, 2.28] \| <0.001 \| \| 2.33 [2.01, 2.69] \| <0.001 \| 1.29 [1.11, 1.51] \| 0.001 \| \|  \| \| \| \| \| \| \| \| | | | | | | | |

*Model 3, adjusting year, demographics, selected risk behaviors and weight management;
Reference group for each model: BMI Level 3 (18.5-24.9 kg/m2); Demographics covariates include age, age, and race/ethnicity; selected risk behaviors included driving after alcohol consumption, suicide attempt, smoking status, current alcohol use, and sexual intercourse.*

| Survey Year: 2005 | | | | | | | |
| --- | --- | --- | --- | --- | --- | --- | --- |
|  | Model 1 | | Model 2 | | | Model 3 | |
|  | Odds Ratio  [95% CI] | p value | | Odds Ratio  [95% CI] | p value | Odds Ratio  [95% CI] | p value |
| BMI Level 1: | 0.60 [0.38, 0.91] | 0.022 | | 0.65 [0.40, 1.01] | 0.065 | 1.13 [0.69, 1.78] | 0.616 |
| BMI Level 2: | 0.60 [0.47, 0.77] | <0.001 | | 0.51 [0.39, 0.66] | <0.001 | 0.89 [0.67, 1.17] | 0.429 |
| BMI Level 4: | 1.61 [1.44, 1.81] | <0.001 | | 1.72 [1.52, 1.94] | <0.001 | 1.11 [0.97, 1.26] | 0.133 |
| BMI Level 5: | 1.95 [1.70, 2.23] | <0.001 | | 2.15 [1.85, 2.50] | <0.001 | 1.19 [1.02, 1.39] | 0.030 |
| \| Survey Year: 2007 \| \| \| \| \| \| \| \| \| --- \| --- \| --- \| --- \| --- \| --- \| --- \| --- \| \|  \| Model 1 \| \| Model 2 \| \| \| Model 3 \| \| \| Odds Ratio  [95% CI] \| p value \| \| Odds Ratio  [95% CI] \| p value \| Odds Ratio  [95% CI] \| p value \| \| BMI Level 1: \| 0.78 [0.49, 1.20] \| 0.286 \| \| 0.78 [0.48, 1.23] \| 0.312 \| 1.29 [0.76, 2.10] \| 0.321 \| \| BMI Level 2: \| 0.68 [0.53, 0.86] \| 0.002 \| \| 0.67 [0.51, 0.86] \| 0.002 \| 1.02 [0.77, 1.33] \| 0.897 \| \| BMI Level 4: \| 1.53 [1.36, 1.72] \| <0.001 \| \| 1.66 [1.46, 1.89] \| <0.001 \| 1.08 [0.95, 1.24] \| 0.245 \| \| BMI Level 5: \| 2.19 [1.91, 2.51] \| <0.001 \| \| 2.38 [2.05, 2.77] \| <0.001 \| 1.38 [1.17, 1.61] \| <0.001 \| \|  \| \| \| \| \| \| \| \| | | | | | | | |

| Survey Year: 2009 | | | | | | | |
| --- | --- | --- | --- | --- | --- | --- | --- |
|  | Model 1 | | Model 2 | | | Model 3 | |
|  | Odds Ratio  [95% CI] | p value | | Odds Ratio  [95% CI] | p value | Odds Ratio  [95% CI] | p value |
| BMI Level 1: | 0.84 [0.56, 1.22] | 0.390 | | 0.92 [0.60, 1.37] | 0.706 | 1.44 [0.92, 2.18] | 0.086 |
| BMI Level 2: | 0.66 [0.52, 0.83] | <0.001 | | 0.66 [0.51, 0.84] | 0.001 | 1.10 [0.84, 1.43] | 0.248 |
| BMI Level 4: | 1.61 [1.44, 1.80] | <0.001 | | 1.72 [1.53, 1.94] | <0.001 | 1.10 [0.97, 1.25] | 0.345 |
| BMI Level 5: | 2.23 [1.94, 2.55] | <0.001 | | 2.36 [2.03, 2.74] | <0.001 | 1.37 [1.17, 1.60] | <0.001 |
|  | | | | | | | |

| Survey Year: 2011 | | | | | | | |
| --- | --- | --- | --- | --- | --- | --- | --- |
|  | Model 1 | | Model 2 | | | Model 3 | |
|  | Odds Ratio  [95% CI] | p value | | Odds Ratio  [95% CI] | p value | Odds Ratio  [95% CI] | p value |
| BMI Level 1: | 0.57 [0.39, 0.81] | 0.003 | | 0.57 [0.38, 0.83] | 0.004 | 0.90 [0.59, 1.34] | 0.620 |
| BMI Level 2: | 0.59 [0.47, 0.74] | <0.001 | | 0.63 [0.49, 0.79] | <0.001 | 0.99 [0.77, 1.26] | 0.912 |
| BMI Level 4: | 1.45 [1.30, 1.62] | <0.001 | | 1.53 [1.35, 1.72] | <0.001 | 1.03 [0.91, 1.17] | 0.659 |
| BMI Level 5: | 1.62 [1.41, 1.86] | <0.001 | | 1.68 [1.45, 1.95] | <0.001 | 1.04 [0.89, 1.22] | 0.598 |

| Survey Year: 2013 | | | | | | | |
| --- | --- | --- | --- | --- | --- | --- | --- |
|  | Model 1 | | Model 2 | | | Model 3 | |
|  | Odds Ratio  [95% CI] | p value | | Odds Ratio  [95% CI] | p value | Odds Ratio [95% CI] | p value |
| BMI Level 1: | 0.95 [0.68, 1.29] | 0.751 | | 0.96 [0.67, 1.34] | 0.803 | 1.66 [1.15, 2.35] | 0.006 |
| BMI Level 2: | 0.69 [0.55, 0.87] | 0.002 | | 0.63 [0.49, 0.80] | <0.001 | 1.00 [0.77, 1.28] | 0.984 |
| BMI Level 4: | 1.60 [1.42, 1.80] | <0.001 | | 1.63 [1.43, 1.85] | <0.001 | 1.12 [0.98, 1.28] | 0.094 |
| BMI Level 5: | 1.96 [1.71, 2.26] | <0.001 | | 1.93 [1.66, 2.24] | <0.001 | 1.19 [1.02, 1.40] | 0.029 |
